# Influence of Aging on Biaxial Flexural Strength and Hardness of Translucent 3Y-TZP

**DOI:** 10.3390/ma13010027

**Published:** 2019-12-19

**Authors:** Nawal M. Moqbel, Majed Al-Akhali, Sebastian Wille, Matthias Kern

**Affiliations:** Department of Prosthodontics, Propaedeutics and Dental Materials, School of Dentistry, Christian-Albrechts University, 24105 Kiel, Germany; malakhali@proth.uni-kiel.de (M.A.-A.); swille@proth.uni-kiel.de (S.W.); mkern@proth.uni-kiel.de (M.K.)

**Keywords:** aging, biaxial flexural strength, hardness, roughness, zirconia

## Abstract

The purpose of this research was to evaluate the influence of aging and surface treatment on surface roughness, biaxial flexural strength (BFS), and Vickers hardness (VHN) of translucent dental zirconia. Half of 80 disc-shaped zirconia specimens (1.2 mm thickness and 12 mm diameter) were aged (group A) in an autoclave for 20 h (134 °C and 0.2 MPa) and the other half were not aged (group N). Specimens were subjected to: no surface treatment (SIN), particle air-abrasion with 50 µm alumina particles at 1 bar (0.1 MPa) and 2.5 bar (0.25 MPa), or polishing down to 1 µm (POL). Specimens were analyzed using X-ray diffraction, laser scanning microscope, BFS, and VHN tests. Three groups (N-SIN, N-POL, and A-POL) showed almost no monoclinic phase. While other groups showed monoclinic phase ratios ranging from 7.5 vol. % ± 2.4 vol. % (N-0.1 MPa) to 41.5 vol. % ± 0.3 vol. % (A-0.1 MPa). Aging and particle air-abrasion increased significantly the BFS, ranging from 720 ± 37 MPa (N-SIN) to 1153 ± 92 MPa (N-0.1 MPa). The hardness was not influenced significantly by aging. A certain amount of monoclinic phase at the surface strengthens the high translucent dental zirconia, while hardness and roughness are not influenced. The pressure of particle air-abrasion showed no influence on the evaluated properties.

## 1. Introduction

Recently, Y-TZP ceramics (yttria-stabilized tetragonal zirconia polycrystals) are the strongest alternative biomedical dental materials for all-ceramic dental restorations because of its esthetic, durability, and availability [1,2,3]. In addition, it is more biocompatible than other ceramics, titanium, and metal alloys, particularly when it is in direct contact with the gingival tissues. Zirconia has three crystalline phases: monoclinic, tetragonal, and cubic phase [4]. Yttrium is added to stabilize zirconia at room temperature in the tetragonal phase [5], accordingly, this transformation is responsible for its high mechanical properties [6].

The mechanical behavior of Y-TZP zirconia might be deteriorated by a spontaneous transformation of the tetragonal phase to monoclinic phase (t→m) when triggered by external stresses such as grinding [7,8] particle air-abrasion [9,10] and hydrothermal aging [11,12] which is called hydrothermal degradation or low temperature degradation (LTD) [13].

LTD initially occurs at the superficial grains, where water is incorporated into zirconia grains by filling oxygen vacancies, and later spreads to the surface increasing its roughness [14,15]. Afterwards, LTD proceeds into the bulk material [15] and affects negatively the density and mechanical properties of Y-TZP structures [2,16,17,18]. LTD can be induced by steam autoclave treatments at increased temperatures (120–140 °C), whereby phase transformation of Y-TZP crystals occurs in the presence of water or steam [18,19,20].

Many studies reported that the real influence of aging of zirconia in an autoclave is not clear. It may positively or negatively influence the mechanical properties of Y-TZP ceramics. First the aging leads to an increase in the mechanical properties, through the transformation toughening mechanism [6,21], then it results in a mechanical deterioration of the material [2,13,22]. The improvement of mechanical properties of zirconia by LTD was reported by several studies [9,12,23], while other studies [24,25] showed that LTD had no effect on the mechanical properties of zirconia.

The strength of zirconia can be influenced also by different surface treatments, such as particle air-abrasion with Al_2_O_3_ particles, silica coating, acid etching and combinations of any of these methods [26,27,28]. Many studies showed that different surface treatments might influence the strength of zirconia ceramic negatively [7,29,30,31]. Different surface damages were created as a result of the surface treatment. These surfaces flaws act as stress concentration areas which later on act as a potential site of crack initiation and propagation [32]. On the other hand, some studies reported an increase in the strength of zirconia with particle air-abrasion treatment due to the created compressive stresses induced by the tetragonal-monoclinic transformation on the surface of zirconia [7,8,33].

Some studies [34,35] reported that the tested polishing systems were not able to induce a tetragonal-monoclinic transformation. While another study [33] showed a significant t→m transformation which resulted in an increase in the biaxial flexural strength of Y-TZP with the polishing treatment.

Monolithic zirconia ceramics used as full-contour ceramic restorations, overcome the problems of veneering porcelain chipping [36,37,38], and have the advantages of restorations requiring less invasive tooth preparations and providing high mechanical and esthetic properties [39].

Dental restorations are exposed in the oral environment to different destructive stimuli such as mastication, the effect of water, and change of temperatures. Currently, there are limited data concerning the influence of aging and different surface treatments on the mechanical properties of translucent 3Y-TZP monolithic zirconia, which might be recommended during the repair of zirconia in the case of chipping or partial fractures under clinical conditions. And some of the studies are contradictory to others. The translucency of zirconia has a strong relationship with its microstructure and chemical compositions [40]. Previous studies showed that the translucency of zirconia is influenced by grain size of Y-TZP ceramics, grain boundary structures, and phase composition [41,42,43]. Different dental manufacturers eliminate or reduced the alumina amount or increased the yttria content to improve the translucency of dental Y-TZP ceramics [44]. Increasing the content of yttria influences negatively the mechanical properties of zirconia [45]. Recently, some literature reported that the co-doping of 0.2 mol % Lanthanum oxide and 0.1 wt. %–0.25 wt. % Al_2_O_3_ in 3Y-TPZ zirconia resulted in a high translucent zirconia with improved mechanical properties [44,46]. It has been reported that there was an increase in the strength of zirconia with the increase in t→m phase transformation following different surface treatments or aging [8,12,33]. In contrast, some studies showed that different surface treatments or aging decrease the strength of zirconia [7,8,30,47], or cause no effect on the mechanical properties of zirconia [34,48,49]. Therefore, the aim of this study was to evaluate the influence of aging and surface treatment on surface roughness, biaxial flexural strength (BFS), and Vickers hardness of high translucent dental zirconia. The tested hypothesis was that both aging and different surface treatments will not influence the BFS or Vickers hardness of high translucent dental zirconia.

## 2. Materials and Methods

**Figure 1:**
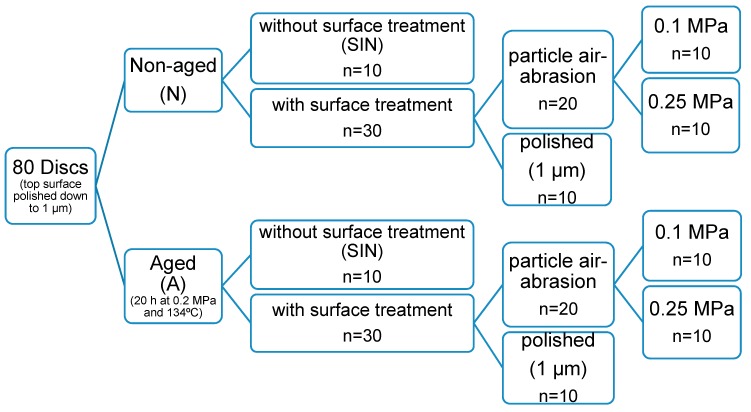
Design of the study for the evaluation of biaxial flexural strength.

The 3Y-TZP ceramic used in this study is a high translucent zirconia (Katana HT10, Batch no.: DPQAY, Kuraray, Tokyo, Japan) containing (ZrO_2_ + HfO_2_ + Y_2_O_3_) > 99.0%, yttrium oxide (Y_2_O_3_) > 4.5%– ≤ 6.0%, hafnium oxide (HfO_2_) ≤ 5.0%, and other oxides ≤ 1.0% with a translucency of 30% according to the manufacturer’s information. Eighty disc-shaped high translucent zirconia with a final diameter of 12 mm, a final thickness of 1.2 mm and shade HT10 were milled and sintered in a sintering oven (Nabertherm GmbH, Bremen, Germany) according to the manufacturer’s instructions (1500 °C, holding time: 2 h). The top side of each disc was polished down to 1 µm grit. After that the specimens were divided according to aging into two main groups, aged (group A) and non-aged (group N). For each group, a different surface treatment was applied: no surface treatment was applied after sintering (group SIN, particle air-abrasion either with 0.1 MPa (group 0.1 MPa), or 0.25 MPa (group 0.25 MPa), and polishing down to 1 µm (group POL) as shown in Figure 1.

### 2.1. Aging

After sintering, half of the specimens were aged (*n* = 40) using an autoclave (CS, WEBECO, Bad Schwartau, Germany) according to ISO standard 13356, at 134 °C, under 0.2 MPa, over a period of 20 h. Then the specimens were cleaned ultrasonically in 99% isopropanol for 3 min. This protocol was used in this study because previous studies showed that aging protocol of 134 °C, 0.2 MPa for 20 h, promotes an extensive t→m phase transformation [50,51]. Thus the aging performed in this study was considered sufficient to observe significant changes induced by different surface treatments.

### 2.2. Surface Treatment

The specimens were subdivided into four sub-groups (*n* = 10), according to the surface treatments applied on the bottom side:

(a) Group SIN: as-sintered (control).

No treatment was applied after sintering.

(b) Group 0. 1MPa: particle air-abrasion treatment using 0.1 MPa.

Particle air-abrasion was done using 50 µm Al_2_O_3_ particles at 0.1 MPa for 15 s at a distance of 10 mm, with Nozzle motion in both lateral directions. Airborne-abrasion was performed using spot blasting unit (P-G 400 K Spot Fine Blasting Unit, Harnish+Rieth, Winterbach, Germany). All specimens were cleaned ultrasonically for 3 min in 99% isopropanol.

(c) Group 0.25MPa: particle air-abrasion treatment using 0.25 MPa.

Same parameters as in 0.1MPa group except the pressure used was 0.25 MPa.

(d) Group POL: Polishing down to 1 µm.

Specimens were polished under cooling water with a diamond grinding disc (Apex CGD, yellow, Buehler, Düsseldorf, Germany) for 10 min to produce a parallel surface, then 1200 and 2500 grit silicon carbide paper (CarbiMet, Buehler, Düsseldorf, Germany) for 140–150 min followed by 3 µm and 1 µm diamond suspensions (MetaDi, Buehler, Düsseldorf, Germany) for 5 min for each. The polishing was performed using a polishing machine (AutoMet 250 and EcoMet 250, Buehler, Düsseldorf, Germany). All specimens were cleaned ultrasonically as described above.

### 2.3. Phase Analysis

Three specimens were selected from each group for X-ray diffraction (XRD) surface analysis to detect the available amount of tetragonal and monoclinic phases. Each specimen was analyzed in a diffractometer (Seifert XRD 3000 PTS, GE GmbH, Munich, Germany) using Cu Kα radiation recorded within the range of 20–40°, a step size of 0.04° and a scanning time per step of 10 s. Voltage and current were 40 kV and 40 mA, respectively. Integrated intensities of the relevant peaks of the diffraction patterns were evaluated using the software Peakfit v.4.12 (Systat Software GmbH, Erkrath, Germany).

The amount of monoclinic phase (X_m_) was calculated using the method developed by Gravie and Nicholson as follows [52]:

X_m_ = (I_M(111)_ + I_M(111_^−^_)_)/(I_M(111)_ + I_M(111_^−^_)_+I_T(111)_),

where M(111) and M(111^−^) represent the intensities of the monoclinic peaks, and T(111) indicates the intensity of tetragonal peak. I_T_ and I_M_ represent the integrated area under the peaks of the tetragonal (111) and monoclinic (111) and monoclinic (111^−^) peaks around 30°, 31°, and 28°, respectively. The volumetric fraction V_m_ was calculated using the following equation [53]:

V_m_ = (1.311 × X_m_)/(1 + 0.311 × X_m_).

### 2.4. Surface Roughness Evaluation

A 3D laser scanning microscope (Keyence VK-X100, Keyence GmbH, Neu-Isenburg, Germany) was used to measure the average roughness value of the surface (Ra) and the mean roughness depth (Rz) for all groups. For each subgroup, three specimens were selected. The measurements were done using a wavelength of 658 nm. The used length for the measured line was 1180 µm and the distance between the separate scans was 0.27 µm.

### 2.5. Biaxial Flexural Strength Test

**Figure 2:**
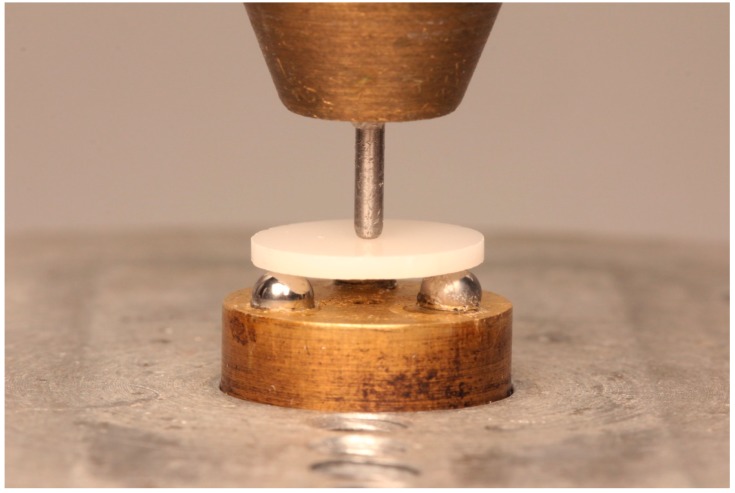
Piston-on-three balls test.

Ten specimens from each subgroup were subjected to biaxial flexural strength test using piston-on-three balls technique in a universal testing machine (Zwick Z010, Zwick, Ulm, Germany). Each disc was placed centrally on three of 3.2 mm diameter steal balls positioned equidistant from each other on a metallic platform with a diameter of 10 mm. Each specimens was loaded by a piston of 1.4 mm diameter at 0.5 mm/min crosshead speed using the universal testing machine as shown in Figure 2.

The biaxial flexural strength was calculated using the following equation [25,54]:

S = −0.2387 × P × ((X−Y)/(d^2^)),

where S is the biaxial flexural strength (MPa); P is the fracture load (N); and d is the specimen disc thickness (mm).

X and Y were determined as follows:

X = (1 + υ) ln (r2/r3)^2^ + [(1 − υ)/2] (r2/r3)^2^,

Y = (1 + υ) [1 + ln (r1/r3)^2^] + (1 − υ) (r1/r3)^2^,

where υ is Poisson’s ratio (0.25); r1 is the radius of support circle; r2 is the radius of loaded area; and r3 is the radius of the specimen.

### 2.6. Vickers Hardness Evaluation

An indentation tester (ZHV10, Zwick, Ulm, Germany) was used to measure the Vickers hardness. A piece from each broken specimen from group SIN and group POL (aged or non-aged) was randomly selected. All particle air-abrasion groups (0.1 or 0.25 MPa) did not undergo hardness test as it is clinically not applicable and the measurements of rougher surfaces were not reliable. The rougher surfaces make it difficult to identify the exact margins of the indentations and to obtain reliable results, as observed with the SIN group measurements. An indentation was made on each specimen on the bottom side using a loading mass of 5 kg and dwell time of 10 s. The measurements of Vickers Hardness number were repeated 4 times at different points, the distance between each point and the other was at least 2 mm. The hardness was computed using the following equation [55,56]:

VHN = 0.1891 × (F/d^2^),

where VHN is the Vickers hardness number, F is the applied load expressed in N, and d is the mean length of the two diagonals of the indentation (mm).

### 2.7. Fracture Pattern

One exemplary sample of each group was selected and coated with a gold layer of 30 nm. Then the fracture pattern was examined using a scanning electron microscope (SEM, XL 30 CP, Philips, Surrey, UK) with an acceleration voltage of 15 kV.

### 2.8. Statistical Analysis

Shapiro-Wilk test was used to inspect the normal distribution of the collected data and then the data were analyzed to determine the effect of aging and surface treatment on the tested variables. One-way analysis of variance (ANOVA) followed by t-test were used for the BFS analysis. While the Kruskal-Wallis and Mann-Whitney-U tests were used to analyze the hardness measurements. The significance level of determination was of *p* ≤ 0.05. All calculations were made with statistical software (IBM SPSS FOR Windows; Version 20.0, IBM SPSS, Inc., Chicago, IL, USA).

## 3. Results

### 3.1. Phase Analysis

**Figure 3:**
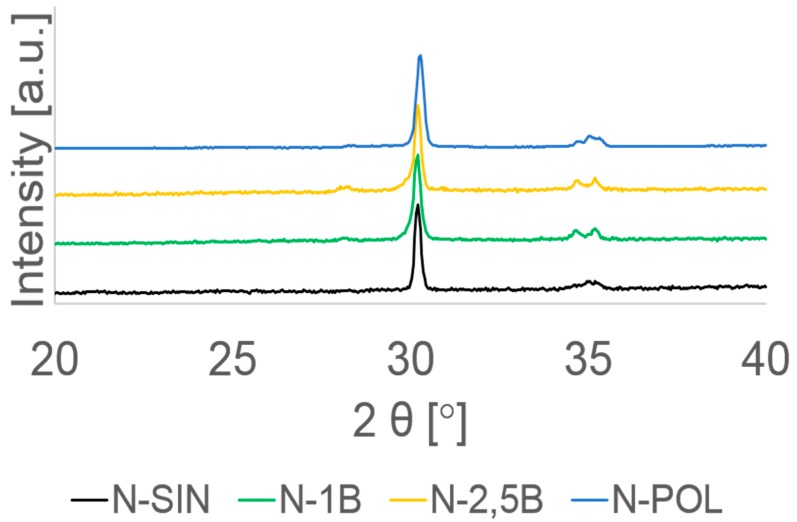
Representative XRD spectra of all non-aged groups with different surface treatments. As sintered (SIN); airborne-abrasion with 50 µm SiO2 at 0.1 MPa (1B); airborne-abrasion with 50 µm SiO2 at 0.25 MPa (2.5B), and polished down to 1 µm (POL).

**Figure 4:**
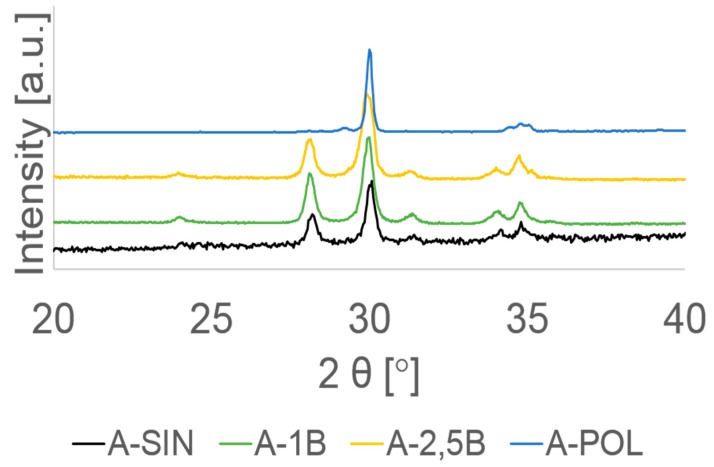
Representative XRD spectra of all aged groups with different surface treatments. As sintered (SIN); airborne-abrasion with 50 µm SiO2 at 0.1 MPa (1B); airborne-abrasion with 50 µm SiO2 at 0.25 MPa (2.5B), and polished down to 1 µm (POL).

XRD analysis showed that both aging and different surface treatment promote different amount of monoclinic phase. The highest values of the monoclinic phase ratio (vol. %) were found within A-SIN, A-0.1MPa, and A-0.25MPa groups. While the N-0.1MPa and N-0.25MPa groups showed a lower monoclinic phase ratio and both polished (N-POL and A-POL) groups and N-SIN group exhibited almost no monoclinic phase (Table 1). A typical XRD spectrum for all groups is shown in Figure 3 and Figure 4.

### 3.2. Surface Roughness Evaluation

The polishing groups either aged or non-aged showed the lowest mean R_a_ and R_z_ values. The descriptive statistics was shown in Table 1.

### 3.3. Biaxial Flexural Strength Test

The mean BFS values increased with aging and particle air-abrasion, as showed in A-SIN, N-0.1 MPa, and N-0.25 MPa groups. Likewise, both A-0.1 MPa and A-0.25 MPa groups showed comparable BFS mean values. While the BFS mean value decreased within both polished groups either aged or non-aged as well as in N-SIN group (Table 2).

### 3.4. Vickers Hardness Evaluation

The Vickers hardness ranged from 1346 HV5 (A-POL) to 1446 HV5 (N-SIN). The aging had no effect on the Vickers hardness. Likewise, with the polishing treatment both polished groups (N-POL and A-POL) reported a lower hardness than both as-sintered groups (N-SIN and A-SIN), as shown in Table 3.

### 3.5. Fracture Pattern

**Figure 5:**
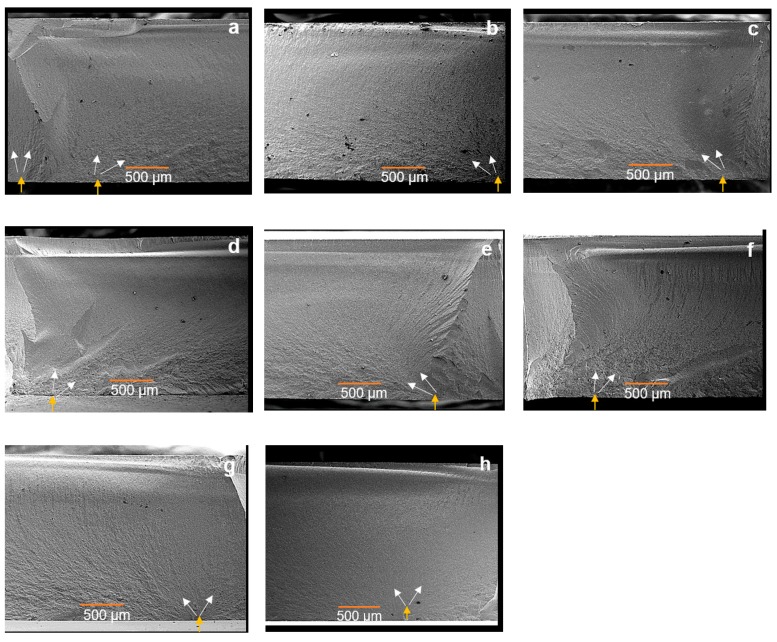
Representative SEM micrographs of typically fractured specimens; (**a**,**b**) as-sintered specimens; (**c**,**d**) airborne-abrasion with 50 µm SiO_2_ at 0.1 MPa; (**e**,**f**) airborne-abrasion with 50 µm SiO_2_ at 0.25 MPa; (**g**,**h**) polished down to 1 µm, exhibiting the origin of failure (yellow arrow on the treated surface). Thin white arrows follow the hackle lines in the direction of crack propagation. In most specimens, the failure was initiated from a surface crack then going deeper through the connected subsurface cracks.

A typical SEM micrograph of a typical samples of each group is shown in Figure 5. In most cases, failure of these samples was initiated from a surface crack. Within the particle air-abrasion treated specimens either aged or not and A-SIN, the SEM micrograph showed many subsurface cracks connected with the surface cracks.

## 4. Discussion

Many studies have been performed to predict the behavior of dental zirconia in the oral environment regarding the aging ability as well as the mechanical properties [2,6,57]. In this concern, the literature shows conflicting results of the effect of aging and surface treatment on the mechanical properties of zirconia.

The aging protocol used in this study was in an autoclave at 134 °C, under 0.2 MPa, over a period of 20 h, which was consistent with previous studies [50,51]. These studies showed that this protocol promotes an extensive t→m phase transformation. Thus, the aging performed in our study should be sufficient to observe significant changes induced by different surface treatments.

In our study, XRD analysis showed a different percentage of phase transformation. The highest monoclinic phase ratio (38.5 vol. % ± 2.8 vol. % to 41.5 vol. % ± 0.3 vol. %) was found within A-0.1MPa, A-0.25MPa and A-SIN groups. These findings show that both aging and particle air-abrasion treatment produced a significant t→m phase transformation. These results are consistent with other studies [8,9,12,24]. In contrast, both non-aged specimens with 0.1 or 0.25 MPa particle air-abrasion showed a lower monoclinic phase ratio (7.5 vol. % ± 2.4 vol. % to 10.4 vol. % ± 1.5 vol. %). These results indicate that the differing pressures during particle air-abrasion treatment produced a comparable amount of monoclinic phase on the surface of zirconia. In addition, both polished groups (N-POL and A-POL) and N-SIN group showed almost no vol. % of monoclinic phase ratio revealing almost no t→m phase transformation on zirconia surface. These results may have been due to neither sintering nor polishing treatment probably promotes monoclinic phase transformation as shown with groups N-SIN and N-POL. These findings also confirm other studies, which showed that polishing produced no t→m phase transformation [34,35,58]. It can be assumed that the previously transformed monoclinic layer (20 µm thicknesses) caused by aging on the surface of zirconia within group A-POL was removed during the polishing treatment.

BFS data demonstrated that groups with monoclinic phase ratio ranging from 7.5 vol. % ± 2.4 vol. % to 41.5 vol. % ± 0.3 vol. % showed the highest mean BFS. However, this increase in the BFS was independent on the percentage of the phase transformation. The increase in the BFS might be explained by the toughening mechanism, as the t→m phase transformation lead to volumetric expansion at a localized area around the superficial defects resulting in a compressive stresses concentrated around these defects and consequently stopping the crack propagation [6,12,52], which need more force to bring the sample to fracture. The SEM micrographs show many subsurface cracks connected with the surface cracks within the particle air-abrasion groups. This indicates, that there were already some microcracks inside the material, which were stopped by phase transformation. This phase transformation induced some compressive stress resulting in a higher BFS as described above. Groups with a lower BFS do not show these many subsurface cracks as there were no microcracks that were stopped by phase transformation. In contrast to the current study, other studies showed that neither aging nor particle air-abrasion affected the strength of 3Y-TZP zirconia [25,48,49,54]. However, only one study [49] investigated the same translucent zirconia.

Regarding the effect of the different used pressure during the particle air-abrasion on the strength of zirconia, our results revealed no significant differences in BFS. In this regard, another study also concluded that there were no significant differences in the BFS of zirconia with particle air-abrasion treatment at different pressures [59].

Likewise, both polished (aged or non-aged) and N-SIN groups showed a reduced BFS, which might be a result of almost no monoclinic phase on the surface of zirconia. In addition, group N-SIN showed a lower BFS in comparison to both polished groups either aged or non-aged. These results may have been due to the different surface roughness of the as-sintered and polished specimens. As the as-sintered specimens showed a rougher surface than the polished specimens, and the surface defects and flaws might later on act as a stress concentration sites, which lead to a significant decrease in the strength of zirconia [29,31]. For this reason the null hypothesis has to be rejected concerning the biaxial flexural strength.

Hardness results of the current study showed that the aging had no effect on the hardness which are in good accordance to the finding of others [60]. In contrast, some studies showed a strong correlation between the reduction of hardness and the increase of monoclinic phase. They reported 41% hardness reduction in femoral head of zirconia with 78% monoclinic phase content [61] and 30% hardness reduction with 48 wt. % ± 1 wt. % monoclinic phase fraction [22]. However, in the current study the monoclinic phase content was 39.9 vol. % ± 0.7 vol. % with the as-sintered aged specimens, which showed no effect on the hardness. One explanation for this might be that the t→m phase transformation percentage was lower than that in previous studies.

Likewise, the findings concerning the effect of polishing treatment on hardness, both as-sintered groups (aged or non-aged) showed a lower hardness than the polished groups (aged or non-aged). Which might be an artefact of the measurement, as the rougher surface of the as-sintered samples makes it difficult to identify the exact margins of the indentations and to obtain reliable results. However, the null hypothesis concerning the hardness is confirmed by the results of the study.

Although the current study showed promising results regarding the mechanical behavior of 3Y-TZP ceramics, it should be considered that the fatigue behavior of this zirconia needs to be investigated under thermal and mechanical loading in aqueous environment over long periods. This will simulate the mechanical loading and thermal conditions in the oral environment, which were not applied in the current study. These and other aspects should be considered in future studies.

## 5. Conclusions

Within the limitations of this laboratory study the following conclusions are drawn:

(1) Both aging and particle air-abrasion demonstrated a significant t→m phase transformation which leads to a significant increase in BFS of high translucent zirconia. While polishing causes no monoclinic phase transformation.
(2) Aging leading to a ratio up to 40 vol. % of monoclinic phase seems to have no effect on the hardness.
(3) The tested different pressures during particle air-abrasion had no influence on the tested mechanical and crystallographic properties.
(4) The positive response of this zirconia to aging and surface treatment seems to provide promising mechanical properties of this material during the repair in case of chipping or partial fractures under clinical conditions.

## Figures and Tables

**Table 1:** Mean and standard deviation (SD) of monoclinic phase ratio of the zirconia measured by XRD and roughness measured by laser scanning microscopy.

Group	Aging	Monoclinic Phase Ratio Mean ± SD (vol. %)	R_a_ Mean ± SD (µm)	R_z_ Mean ± SD (µm)
SIN	Non-aged (N)	0.0 ± 0.0	0.449 ± 0.096	2.321 ± 0.422
	Aged (A)	39.9 ± 0.7	0.502 ± 0.055	2.434 ± 0.221
0.1 MPa	Non-aged (N)	7.5 ± 2.4	0.531 ± 0.051	2.738 ± 0.176
	Aged (A)	41.5 ± 0.3	0.434 ± 0.032	2.241 ± 0.136
0.25 MPa	Non-aged (N)	10.4 ± 1.5	0.528 ± 0.020	2.845 ± 0.092
	Aged (N)	38.5 ± 2.8	0.485 ± 0.044	2.480 ± 0.150
POL	Non-aged (N)	2.1 ± 0.6	0.006 ± 0.001	0.034 ± 0.012
	Aged (A)	2.1 ± 0.5	0.002 ± 0.001	0.014 ± 0.003

**Table 2:** Mean and standard deviation of biaxial flexural strength (BFS) (MPa) of all tested groups.

Groups	Aging	
	Non-Aged Groups	Aged Groups
	Mean ± SD	Mean ± SD
SIN	720 ± 37 C, α	1064 ± 27 A, β
0.1 MPa	1153 ± 92 A, α	1110 ± 76 A, α
0.25 MPa	1137 ± 89 A, α	1105 ± 74 A, α
POL	894 ± 96 B, α	888 ± 86 B, α

Within the same column, mean with the same capital letter are not statistically different (*p* > 0.05); within the same row, mean with the same Greek letter are not statistically different *p* > 0.05.

**Table 3:** Median, mean and standard deviation of hardness (HV5).

Groups	Aging			
	Non-Aged Groups		Aged Groups	
	Median	Mean ± SD	Median	Mean ± SD
SIN	1446 Aα	1413 ± 127	1436 Aα	1428 ± 98
POL	1347 Bα	1340 ± 21	1346 Bα	1346 ± 18

Within the same column, mean with the same capital letter are not statistically different (*p* > 0.05); within the same row, mean with the same Greek letter are not statistically different *p* > 0.05.
